# Morphological identification of *Lucilia sericata*, *Lucilia cuprina* and their hybrids (Diptera, Calliphoridae)

**DOI:** 10.3897/zookeys.420.7645

**Published:** 2014-06-25

**Authors:** Kirstin A. Williams, Martin H. Villet

**Affiliations:** 1 Entomology Department, Durban Natural Science Museum, Durban, South Africa; 2 Southern African Forensic Entomology Research Laboratory, Department of Zoology and Entomology, Rhodes University, Grahamstown, 6140 South Africa

**Keywords:** Greenbottle blowflies, keys, morphology, discriminant analysis

## Abstract

Hybrids of *Lucilia sericata* and *Lucilia cuprina* have been shown to exist in previous studies using molecular methods, but no study has shown explicitly that these hybrids can be identified morphologically. Published morphological characters used to identify *L. sericata* and *L. cuprina* were reviewed, and then scored and tested using specimens of both species and known hybrids. Ordination by multi-dimensional scaling indicated that the species were separable, and that hybrids resembled *L. cuprina*, whatever their origin. Discriminant function analysis of the characters successfully separated the specimens into three unambiguous groups – *L. sericata*, *L. cuprina* and hybrids. The hybrids were morphologically similar irrespective of whether they were from an ancient introgressed lineage or more modern. This is the first evidence that hybrids of these two species can be identified from their morphology. The usefulness of the morphological characters is also discussed and photographs of several characters are included to facilitate their assessment.

## Introduction

The use of maggot debridement therapy (MDT) in South Africa has gained interest in the past decade (Williams et al. 2008, Du Plessis and Pretorius 2011). The identification of the maggots used for this therapy remains an issue, as most medical doctors are not adequately trained in entomology to correctly identify the flies (Williams et al. 2008, Tantawi et al. 2010). *Lucilia sericata* is the most commonly used species (Sherman et al. 2000) but it is often misidentified as *Lucilia cuprina*. These two species are also used in forensic entomology (Louw and van der Linde 1993, Smith and Wall 1997, Anderson 2000, Oliva 2001, Clark et al. 2006, Day and Wallman 2006) and *Lucilia cuprina* is the species most often responsible for sheep strike – myiasis of sheep by the maggots of this fly (Hepburn 1943, Ullyett 1945, Vogt and Woodburn 1979, Heath and Bishop 2006), but *Lucilia sericata* is responsible for sheep strike in northern Europe where *Lucilia cuprina* is absent (Rose and Wall 2011). Correct identification of these flies is thus vitally important for these three fields.

Several identification keys have been produced either specifically for *Lucilia sericata* and *Lucilia cuprina*, or for larger suites of Luciliinae or Calliphoridae that included these two species (Waterhouse and Paramonov 1950, Rognes 1980, 1994, Dear 1986, Holloway 1991, Wallman 2001, Whitworth 2006, 2010), but several of the diagnostic characters are sometimes omitted while others are included that are less reliable or difficult to observe. Although both species occur worldwide, some of the differences between the character suites in these studies may arise from considering samples from relatively limited geographical regions. The first aim of this study was to consider the value of the published characters based on a sample of specimens from across the world.

A complicating factor is the known and widespread existence of natural hybrids of these species (Stevens et al. 2002, Wallman et al. 2005, Tourle et al. 2009, DeBry et al. 2010, Williams and Villet 2013), which has been established by molecular methods. Tourle et al. (2009) developed a semi-quantitative morphological index for discriminating *Lucilia sericata* and *Lucilia cuprina*, and it provides some evidence that their hybrids might also be morphologically distinguishable. Specifically, genetically identified hybrid specimens tended to show more extreme index values than either parent species. The index incorporated six characters: femur colour; the numbers of paravertical setulae, scutellar hairs and humeral hairs; the pattern of the postoccular microtrichial pile; the length of the sternal hairs of males; and the position of the inner vertical seta of females. The second aim of this study was to determine if hybrid specimens can in fact be determined from their morphology.

## Materials and methods

Twenty-four specimens of *Lucilia sericata*, *Lucilia cuprina* and their hybrids (Table 1) were chosen from specimens that had been sequenced for 28S, COI and Per genes (Williams and Villet 2013). These specimens were chosen to include geographically diverse locations including Egypt, France, Germany, Japan, Namibia, South Africa, Thailand, the United States of America and Zimbabwe.

**Table 1.** Specimens previously identified by molecular markers (Williams and Villet 2013) used in the morphological analyses. (*hybrids).

Species	Specimen	Country of origin
*Lucilia cuprina*	C_EGT_01	Egypt - Alexandria
*Lucilia cuprina*	C_SA_BFN_01	South Africa – Bloemfontein
*Lucilia cuprina*	C_SA_BFN_02	South Africa – Bloemfontein
*Lucilia cuprina*	C_SA_BRT_01	South Africa – Britstown
*Lucilia cuprina*	C_SA_BRT_02	South Africa – Britstown
*Lucilia cuprina*	C_SA_DBN_12	South Africa – Durban
*Lucilia cuprina	C_SA_DBN_01	South Africa – Durban
*Lucilia cuprina	C_SA_DBN_06	South Africa – Durban
*Lucilia cuprina	C_SA_NEL_01	South Africa – Nelspruit
*Lucilia cuprina	C_SA_NEL_02	South Africa – Nelspruit
*Lucilia cuprina	C_THA_03	Thailand – Chiang Mai
*Lucilia cuprina	C_ZIM_02	Zimbabwe – Matobos
*Lucilia sericata*	S_FRC_02	France – Montferrier-Sur-Lez
*Lucilia sericata*	S_GER_01	Germany – Kempen
*Lucilia sericata*	S_JPN_04	Japan – Iwate
*Lucilia sericata*	S_NAM_01	Namibia – Possession Island
*Lucilia sericata*	S_NAM_02	Namibia – Possession Island
*Lucilia sericata*	S_SA_CT_01	South Africa – Cape Town
*Lucilia sericata*	S_SA_CT_05	South Africa – Cape Town
*Lucilia sericata*	S_SA_GHT_01	South Africa – Grahamstown
*Lucilia sericata*	S_SA_GHT_02	South Africa – Grahamstown
*Lucilia sericata*	S_SA_PTA_02	South Africa – Pretoria
*Lucilia sericata*	S_SA_WTB_02	South Africa – Witbank
*Lucilia sericata*	S_USA_01	United States of America – Michigan

A total of 18 distinguishing morphological characteristics of adults of *Lucilia sericata* and *Lucilia cuprina* (Table 2) were obtained by reviewing several published sources (Waterhouse and Paramonov 1950, Rognes 1980, 1994, Dear 1986, Holloway 1991, Wallman 2001, Tourle et al. 2009, Whitworth 2006, 2010). Three characters referred to the male genitalia and three characters were specific to females. The males’ characters could not be viewed without dissecting the specimens and because the majority of the genetically-identified specimens were female (Williams and Villet 2013), it was decided to include only females in the analysis. This reduced the number of characters to 15. Photographs of the specimens were taken using a Nikon D800 camera with a 105 mm lens and 124 mm extension to show several of the characters.

**Table 2.** Published morphological characters used to distinguish specimens of *Lucilia sericata* and *Lucilia cuprina*.

Character	Lucilia sericata	Lucilia cuprina	Analysis	
			MDS	DFA
**General**				
Number of paravertical setulae or occipital bristles (Waterhouse and Paramonov 1950, Dear 1986, Holloway 1991, Rognes 1994, Whitworth 2006, 2010)	Usually 2+2 but up to 8+8 (not always equal numbers i.e. can be 1+2 etc.)	1+1	yes	no
Shape of postocular microtrichial pile on vertex (viewed obliquely from behind) (Holloway 1991)	Boundary between pale and dark areas not straight or sharply defined	Boundary straight and sharply defined	no	no
Width of the frontal stripe (frontal vitta) (Waterhouse and Paramonov 1950, Rognes 1980, 1994)	Twice as wide as a parafrontal (fronto-orbital) plate	As wide as a parafrontal (fronto-orbital) plate	yes	yes
Colour of the frontoclypeal membrane (Waterhouse and Paramonov 1950, Wallman 2001)	Light brown	Dark brown to black	yes	yes
Second pair of presutural acrostichals (Waterhouse and Paramonov 1950)	Extend at least as far as insertions of the first pair of postsutural acrostichals	Do not extend to first pair of postsutural acrostichals	yes	no
Number of setulae on ‘quadrat’ between discal setae and anterior margin of scutellum (Holloway 1991)	35–55	15–25	yes	yes
Bristles on the scutellum (Waterhouse and Paramonov 1950)	Dorsal bristles distinctly smaller than lateral hairs	Dorsal bristles slightly smaller than or equal to lateral hairs	no	no
Number of hairs on the posterior slope of the humeral callus behind the basal setae (Waterhouse and Paramonov 1950, Rognes 1994, Whitworth 2006)	6–8	0–4	yes	yes
Number of hairs on the edge of the notopleuron behind the posterior notopleural seta (Waterhouse and Paramonov 1950, Rognes 1994, Whitworth 2006)	8–16	2–5	yes	yes
Metasternal area – sclerite midventrally between middle and hind coxae (Rognes 1994, Wallman 2001, Whitworth 2006)	Hairy	Bare	no	no
Colour of the fore femora (Waterhouse and Paramonov 1950, Dear 1986, Wallman 2001)	Dark metallic blue to black or dark brown	Metallic green	yes	yes
Contour of the last abdominal tergite (Waterhouse and Paramonov 1950)	Irregular depressions	Generally smooth	no	no
**Females**				
Distance between the outer and inner vertical setae of females (Holloway 1991)	Equal to 0.5–0.7 distance between prevertical and inner vertical setae	Equal to the distance between prevertical and inner vertical setae	yes	no
Size of the angle formed by the inner vertical seta relative to the prevertical and outer vertical setae of females (Holloway 1991)	Obtuse	Right angle	yes	no
Extent of metallic sheen on parafrontal sclerites of females (Holloway 1991)	From vertex barely to base of upper orbital seta and not enclosing bases of any frontal setae	From vertex almost to base of lower orbital seta and enclosing bases of 1 or 2 frontal setae	yes	yes
**Males**				
Shape of apical halves of cerci (Waterhouse and Paramonov 1950, Holloway 1991)	Broad and tapering	Slender and parallel	no	no
Shape of apical halves of surstyli (Waterhouse and Paramonov 1950, Rognes 1980, Holloway 1991)	Curved and broad	Straight and slender	no	no
Form of apical setae of cerci (Holloway 1991)	Long and wavy	Minute and straight	no	no

Each specimen was scored against the 15 characters (Table 2). Each character was then evaluated for its effectiveness in discriminating between the species and its practical value for identification, first univariately and qualitatively, and then multivariately and quantitatively using non-metric multi-dimensional scaling (MDS) in PAST3 (Hammer et al. 2001) using a Manhattan distance metric because of the mixed data forms in the character state matrix.

To explore the diagnosibility of the hybrids, a discriminant function analysis (DFA) was performed using PAST3 (Hammer et al. 2001) on the scored character matrix to determine which characters were most influential in identifying the species. Four of the 15 characters (shape of postocular microtrichial pile, hairiness of metasternal area, contour of the last abdominal tergite, bristles on the scutellum; Table 2) were either not easily visible or the hairs were broken or missing in at least half of the specimens and were therefore excluded from the DFA. Another four of the characters showed no variation within species and therefore had to be excluded from the DFA, which therefore included only seven characters (Table 2). The hybrid specimens were treated as a separate group in this analysis, but the introgressed and modern hybrids were not separated.

## Results

### Univariate assessment of characters

**Figure 1.**
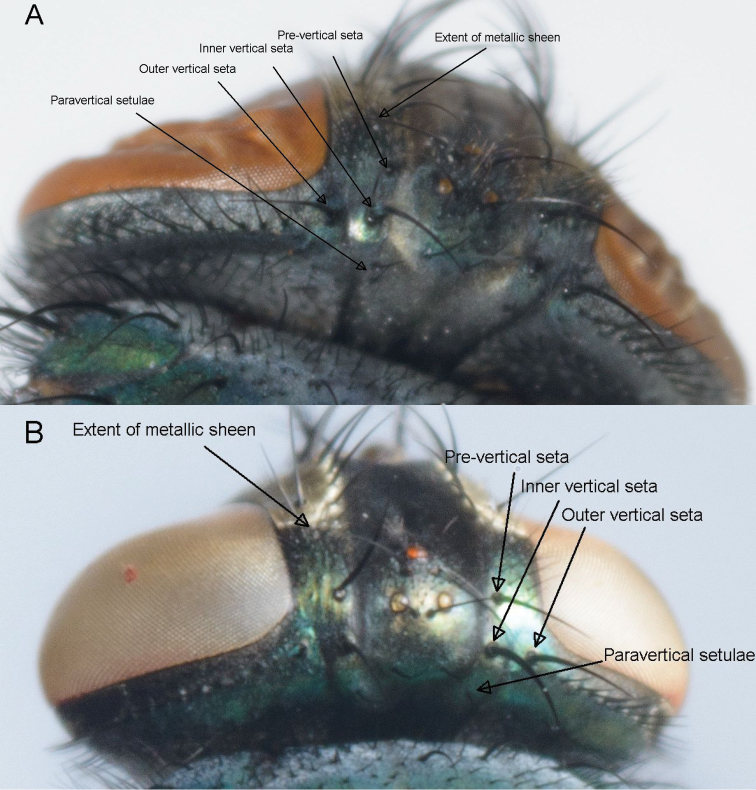
Paravertical setulae, distance between the outer and inner vertical setae, the size of the angle at the inner vertical triangle and extent of metallic sheen on parafrontal sclerites. *Lucilia sericata* (**A**) and *Lucilia cuprina* (**B**).

**The number of paravertical setulae** or occipital bristles (Table 2; Figure 1). This character was relatively consistent and reliable, but it is not easily viewed and scored if the specimens have been kept in ethanol. The hybrid specimens all keyed out as *Lucilia cuprina*. This character was left out of the DFA analysis due to lack of variation within *Lucilia cuprina*.

**The shape of the postocular microtrichial pile** on the vertex (Table 2) (Holloway 1991) is a difficult character to see when the specimens have been stored in ethanol because the microtrichia are not visible unless the specimen is dry, and even then the microtrichia sometimes appear to be absent. Due to the difficulty in viewing and scoring this character, it was eventually left out of all further analyses.

**The relative positions of the three vertical setae** (Table 2; Figure 1) that form a triangle on either side of the ocellar triangle in females (Holloway 1991) is a reliable character that consistently separated the two species. This character was excluded from the DFA because it did not show variation within taxa but was included in the MDS analysis. The hybrid specimens consistently keyed out as *Lucilia cuprina*.

**The angle formed by the three vertical setae** (Table 2; Figure 1). This character is consistent and easily seen even if the setae have fallen out as they have sockets, which are easily visible. Due to lack of variation within species and the hybrids being identified as *Lucilia cuprina*, this character was also excluded from the discriminant function analysis but it was included in the MDS analysis.

**The extent of the metallic sheen on the parafrontal sclerites of females** (Table 2 and Suppl. material 1; Figure 1). This character is easier to observe in dried specimens than ethanol-preserved specimens and there is some variation. The division between the two species is not absolute – there is some overlap within this character but it was not specific to the hybrids. It was included in both the DFA and MDS analyses.

**Figure 2.**
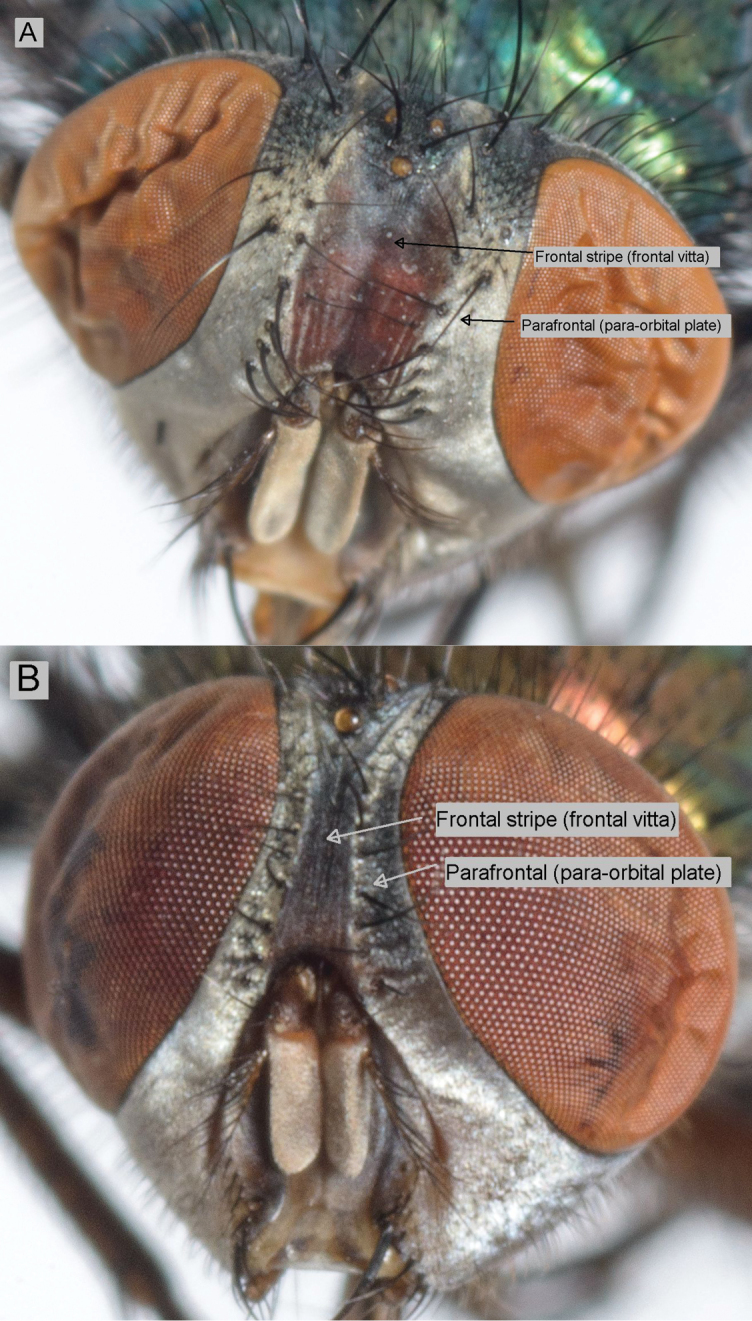
Frontal stripe – *Lucilia sericata* (**A**) and *Lucilia cuprina* (**B**).

**The relative width of the frontal stripe** (frontal vitta) (Table 2 and Suppl. material 1; Figure 2). Waterhouse and Paramonov (1950) suggested that this character was more reliable in males than females. We found that the width varied from being equal to the parafrontal to being more than twice the width in both species. The hybrids were not distinguishable from *Lucilia cuprina*. This character was included in the MDS and the DFA analyses.

**Figure 3.**
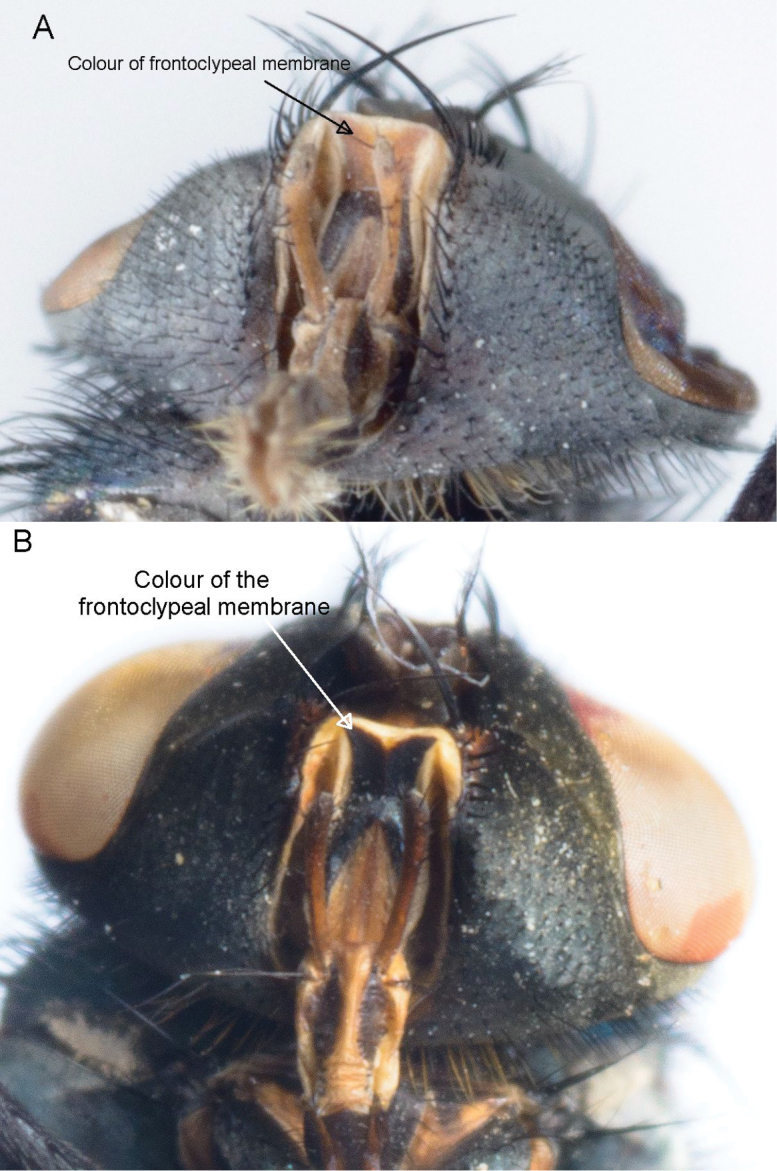
Colour of the frontoclypeal membrane. *Lucilia sericata* (**A**) and *Lucilia cuprina* (**B**).

**The colour of the frontoclypeal membrane** (Table 2 and Suppl. material 1; Figure 3). It was not always easily visible if the proboscis was not extended but it could usually be viewed by either manipulating the proboscis or viewing the specimen from a lateral angle (Waterhouse and Paramonov 1950). The hybrid specimens were not distinct from *Lucilia sericata* or *Lucilia cuprina*.

**The length of the second pair of presutural acrostichals** (Table 2) is a character that is easier to see in well-preserved specimens (Waterhouse and Paramonov 1950). This character is not scorable if the bristles are broken or have fallen out. It was left out of the analyses because it does not show any intraspecies variation.

**Figure 4.**
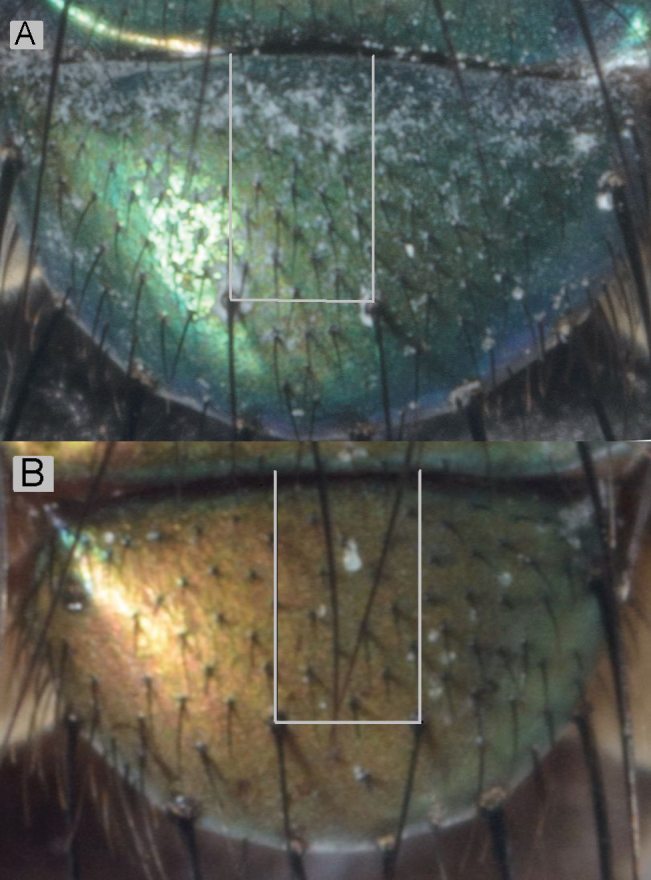
Number of setae on ‘quadrat’ between the anterior margin and discal setae on the scutellum. *Lucilia sericata* (**A**) and *Lucilia cuprina* (**B**).

**The number of setae on the scutellum** (Table 2 and Suppl. material 1; Figure 4) in the ‘quadrat’ demarcated by the discal setae and the anterior margin of the scutellum represents the axis in the discriminant analysis that separated *Lucilia sericata* and *Lucilia cuprina* (Holloway 1991). This character can be used even when the setae have fallen out because they have sockets that are visible and can be counted. There was overlap in the number of setae between the two species, but generally *Lucilia cuprina* had obviously fewer setae. The number of setae in the hybrids was not obviously different from either of the pure species. This overlap may be as a result of the challenge of counting the setae as they are not in straight rows.

**The length of the bristles on the scutellum** (Table 2 and Suppl. material 1) describes the length of the hairs between the two anterior bristles on the lateral margin of the scutellum in relation to the length of the hairs on the dorsal surface of the scutellum (Waterhouse and Paramonov 1950). This character was not easy to use as the hairs were broken or had fallen out in half of the specimens and therefore it was left out of the analyses.

**Figure 5.**
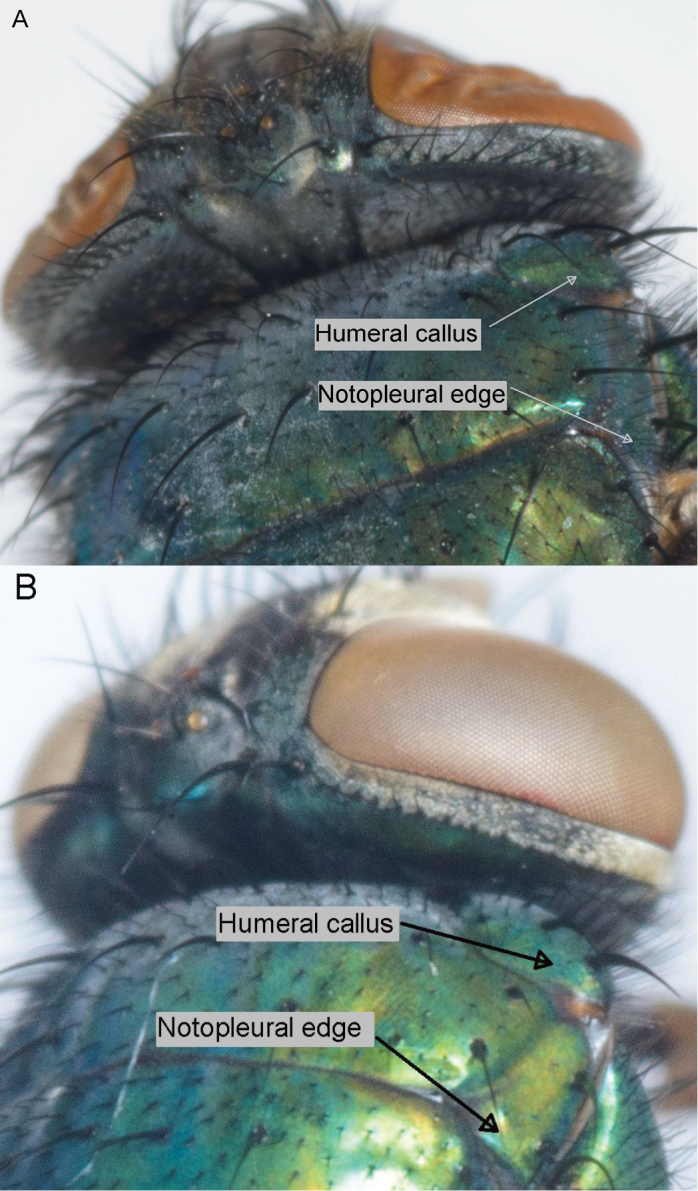
Posterior slope of the humeral callus behind the basal setae and the posterior edge of notopleuron behind the posterior notopleural seta. *Lucilia sericata* (**A**) and *Lucilia cuprina* (**B**).

**The hairiness of the posterior slope of the humeral callus** (Table 2 and Suppl. material 1; Figure 5) behind the basal setae is a reliable character in separating *Lucilia sericata* and *Lucilia cuprina* even though there is variation within species in the number of hairs. The hybrids tended to have more hairs than the pure *Lucilia cuprina* specimens, but there was still overlap in the numbers of hairs between the hybrids and pure *Lucilia cuprina*.

**The number of hairs on the edge of the notopleuron** (Table 2 and Suppl. material 1; Figure 5). Both the hairs on the notopleuron and the humeral callus are relatively easy to observe although ethanol-preserved specimens need to be dried so that the small hairs are visible. It is another reliable character in separating *Lucilia sericata* from *Lucilia cuprina* despite variation in the number of hairs within species. The hybrids showed no discernable difference in numbers of hairs from *Lucilia cuprina*.

**The hairs on the metasternal area** (Table 2), which is the sclerite mid-ventrally between the middle and hind coxae, are exceedingly difficult to view if the legs are not set appropriately to facilitate this.. All of the specimens that we examined were preserved in ethanol and it was not easy to view the metasternal area and this character was therefore not analysed.

**The colour of the fore femora** (Table 2 and Suppl. material 1) has long been used as a character to identify *Lucilia sericata* and *Lucilia cuprina* (Ullyett 1945). It is a controversial character as it varies according to when the flies were killed, if the adults were fully matured and if the specimens were fouled or not during collection and thus is subject to personal interpretation. The hybrids keyed out as *Lucilia cuprina*. Due to the variation in this character it was included in the DFA.

**The contour of the last abdominal tergite** (Table 2) is applicable only to dried specimens (Waterhouse and Paramonov 1950) as it relies on the hardness of the tergite. It was therefore not a character that could be used in our analyses as all our specimens were ethanol-preserved. It was excluded from the analyses and is probably unreliable even in dried specimens because it relies on the preservation of the specimen and how it is pinned, which affects the contour of the last abdominal tergite.

### Multivariate assessments of characters

**Figure 6.**
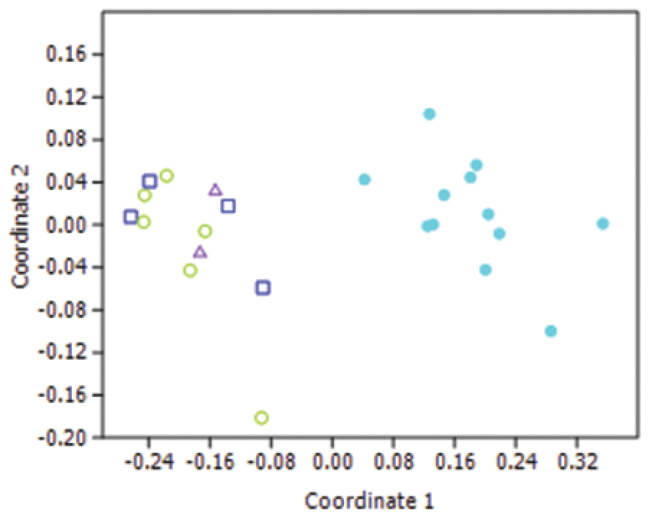
Non-metric Multi-Dimensional Scaling plot using a Manhattan distance metric using 11 characters. Light blue solid circles = *Lucilia sericata*, Green open circles = *Lucilia cuprina*, dark blue squares = introgressed hybrids, purple triangles = modern hybrids.

Superficially, the hybrid specimens were identified as *Lucilia cuprina* when keyed out using any of the published keys. There were no obvious differences in the morphology of the hybrids. When the characters were analysed using MDS, the hybrid specimens were not separated from the *Lucilia cuprina* specimens (Figure 6).

**Figure 7.**
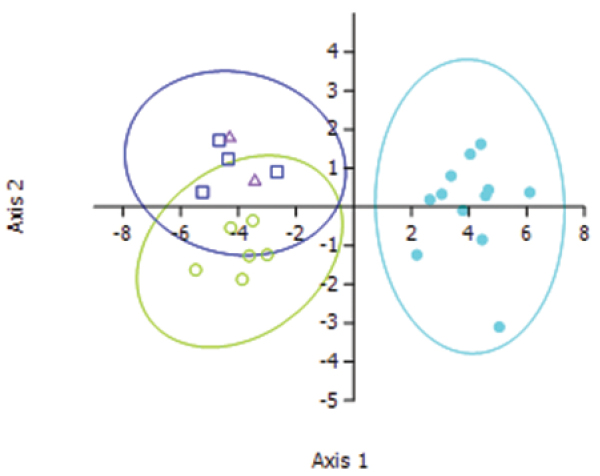
Ordination plot of the first two roots of the discriminant function analysis using seven characters. Ellipses represent 95% confidence regions. Light blue solid circles = *Lucilia sericata*, Green open circles = *Lucilia cuprina*, dark blue squares = introgressed hybrids, purple triangles = modern hybrids.

However, the ordination plot of the DFA (Figure 7) clearly shows three groups – *Lucilia sericata*, *Lucilia cuprina* and hybrids. The most influential characters were the number of setae on the scutellum (Root 1) and the number of hairs on the humeral callus (Root 2) (Table 3). It is not obvious in the morphology that there is a difference between the pure and hybrid strains, but statistically one can separate the hybrids from the pure *Lucilia cuprina* specimens.

**Table 3.** Eigen vectors and values for the first two roots of the discriminant function analysis.

Character	Root 1	Root 2
Number of setulae on ‘quadrat’ demarcated by discal setae and anterior margin of scutellum	**1.5822**	0.0324
Number of hairs on edge of notopleuron behind posterior notopleural seta	0.5576	0.3300
Number of hairs on posterior slope of humeral callus behind basal setae	0.4216	**0.9066**
Colour of fore femora	0.2591	-0.2023
Relative width of frontal stripe (frontal vitta)	0.1551	0.0104
Extent of metallic sheen on parafrontal sclerites of females	0.0519	-0.0697
Colour of frontoclypeal membrane	-0.1551	-0.0104
Eigenvalue	18.5560	0.7406

## Discussion

### Assessment of characters

Due to the greater number of female flies in the molecular study from which we chose our specimens, we did not include any males. Therefore the male genitalia characters are not discussed in detail. It is not possible to properly view the male genitalia without dissecting them and this is not ideal for non-entomologists such as medical doctors who are using these flies for MDT as one needs experience to dissect out the genitalia. It is possible to correctly identify these flies without using the male genitalia by using the other characters described in Table 2.

### Geographical variation

Holloway (1991) suggested that the characters that she described were specifically for *Lucilia sericata* and *Lucilia cuprina* from New Zealand and that they might not apply to specimens from other parts of the world. This does not seem to be the case, as the flies examined in this study are from several different countries around the world (Table 1) and the characters described (excluding the male genitalia) were useful in identifying these two species and their hybrids.

### Identifying hybrids

The DFA unambiguously separated the *Lucilia cuprina* specimens from the hybrids and it was statistically significant. This was not noted in previous studies where hybrids were identified only through molecular techniques (Stevens et al. 2002, Wallman et al. 2005, Tourle et al. 2009, DeBry et al. 2010, Williams and Villet 2013). Examination of the number of hairs on the scutellum, humeral callus and notopleuron show a consistent difference that separates these groups. The first two characters were included in the morphological index designed by Tourle et al (2009), which explains the trend found in their results.

The introgressed and modern hybrids were not separated in the DFA ordination plot (Fig. 6).

## Conclusion

Introgressed and modern hybrids of *Lucilia sericata* and *Lucilia cuprina* can be statistically recognized using the characters described in this paper.

Four of the characters were consistently successful at separating *Lucilia sericata* and *Lucilia cuprina* (number of paravertical setulae or occipital bristles, distance between the outer and inner vertical setae of females, size of the angle at the inner vertical in triangle joining pre-, outer and inner vertical setae of females, second pair of presutural acrostichals) with little variation within the characters. The number of setae on the scutellum and the number of hairs on the humeral callus and notopleuron are also useful characters although they did show variation within species. It is advisable to use a combination of several characters to identify these two species as no single character was sufficient to separate *Lucilia sericata* and *Lucilia cuprina*.

## Supplementary material

Supplementary material 1 Character-taxon matrix doi: 10.3897/zookeys.420.7645.app1 Data type: Species data Explanation note: Character-taxon matrix used in the MDS and DFA analyses This dataset is made available under the Open Database License (http://opendatacommons.org/licenses/odbl/1.0/). The Open Database License (ODbL) is a license agreement intended to allow users to freely share, modify, and use this Dataset while maintaining this same freedom for others, provided that the original source and author(s) are credited.Kirstin A. Williams, Martin H. Villet
